# A 2D Magneto-Acousto-Electrical Tomography Method to Detect Conductivity Variation Using Multifocus Image Method

**DOI:** 10.3390/s18072373

**Published:** 2018-07-21

**Authors:** Ming Dai, Xin Chen, Tong Sun, Lingyao Yu, Mian Chen, Haoming Lin, Siping Chen

**Affiliations:** 1 School of Biomedical Engineering, Health Science Center, Shenzhen University, Shenzhen 518060, China; daily2020@sina.com (M.D.); sunson08@163.com (T.S.); mian.chen@szu.edu.cn (M.C.); linhaomail@163.com (H.L.); 2 Guangdong Key Laboratory for Biomedical Measurements and Ultrasound Imaging, Shenzhen 518060, China; 3 National-Regional Key Technology Engineering Laboratory for Medical Ultrasound, Shenzhen 518060, China; 4 Guangdong Institute of Medical Instruments, Guangzhou 510000, China; lingyaoyu01@163.com

**Keywords:** multifocus imaging, conductivity distribution, chirp signal excitation, digital signal processing

## Abstract

As magneto-acoustic-electrical tomography (MAET) combines the merits of high contrast and high imaging resolution, and is extremely useful for electrical conductivity measurement, so it is expected to be a promising medical imaging modalities for diagnosis of early-stage cancer. Based on the Verasonics system and the MC600 displacement platform, we designed and implemented a MAET system with a chirp pulse stimulation (MAET-CPS) method and a focal probe was utilized for stepscan focus excitation to enhance the imaging resolution. The relevant experiments were conducted to explore the influence of excitation positions of the single-focus point, and the effect of the excitation position on the amplitudes of the conductivity variation was clearly demonstrated. In order to take advantage of the merits of multifocus imaging, we firstly proposed a single focus MAET system with a chirp pulse stimulation (sfMAET-CPS) method and a multifocus MAET system with a chirp pulse stimulation (mfMAET-CPS) method for high-resolution conductivity imaging, and a homogenous gelatin phantom with a cuboid-shaped hole was used to investigate the accuracy of mfMAET-CPS. Comparative experiments were carried out on the same uniform phantom by the sfMAET-CPS and the mfMAET-CPS, respectively. The results showed that: (1) the electrical conductivity distributions of the homogenous phantom with a cuboid-shaped hole were detected by the sfMAET-CPS but were easily affected by the focal point, which demonstrated that the sfMAET-CPS had a low imaging resolution. (2) Compared with the sfMAET-CPS, the imaging effect of the mfMAET-CPS was much better than that of the sfMAET-CPS. (3) A linear interpolation algorithm was used to process the 2D conductivity distribution; it increased the smoothness of the conductivity distribution and improved the imaging effect. The stepscan focus excitation and the linearly frequency-modulated theory provide an alternative scheme for the clinical application of MAET.

## 1. Introduction

Electrical conductivity shows good contrast in the human body [1,2] and the variation of the electrical conductivity is a typical characteristic of most tumors in the early stages [3] due to the change of conductivity being able to identify the physiological or pathological state [3,4]. Thus, electrical conductivity tomography for biological tissue is hopefully likely to become one of the most effective tumor assessment modalities for the early diagnosis of cancer [3], and recently, health information relating the physiological and pathological condition of biological tissues has been obtained by detecting electrical conductivity [4], whereby the main measurement means is electrical impedance tomography (EIT) [5]. Despite having the strength of high contrast, EIT is limited by the quantity of the electrodes [3] and the ill-posed nature of the inverse problem [6], which means it barely achieves a high imaging resolution [7]. Combining the magnetic resonance current density imaging and the EIT technique, magnetic resonance electrical impedance tomography provides cross-sectional conductivity images of an object’s high spatial resolution impedance information [8]. However, this relies on magnetic resonance imaging, which has the weakness of long imaging time and high cost [9]. In magnetoacoustic tomography, the target sample is placed in a uniform magnetic field and an electrical current density inside the sample is generated either by means of induction using a stimulating coil or by injection through a pair of stimulating electrodes [10]. In magnetoacoustic tomography with current injection, a current is injected into imaging body and the diffuse distribution of the current within the imaging body reduces the spatial resolution. Coils are widely used as an excitation source in magnetoacoustic tomography with magnetic induction [11,12], but there exists a dynamic alternating magnetic field in tissue that affects the current within the target sample and thus a high-strength transient excitation magnetic field is required. To overcome the limitations of the aforementioned traditional medical imaging technologies, magneto-acoustic-electrical tomography (MAET) [9,13] combines the advantages of the magnetic, acoustic, and electric fields and merges the superiorities of both traditional EIT and ultrasonography [10]. The cost is relatively low due to the use of static magnetic field [14], and the MAET signal collected by the electrodes is easily detected and processed [14,15], so the MAET is widely and intensively investigated worldwide and has a high possibility of widespread application [1], which has aroused strong interest among medical imaging investigators. For instance, Roth presented a series of methods for MAET and proved several limitations of the methods [16]. Kunyansky has researched the inversion procedure of MAET and proposed a variety of methods to reconstruct the conductivity [17]. In [18], detection of the MAET signal using a Helmholtz coil pair configuration was evaluated. To improve the detection sensitivity of the MAET signal from a homogenous phantom, dual-frequency ultrasound has been used to produce the MAET signal at different frequencies through the ultrasound radiation force mechanism [19]. Liu et al. designed a MAET detection apparatus for detecting the interface of conductivity variations, and numerical simulations, and conductivity imaging experiments were performed on pork tissue and a low-conductivity gelatin imitation having similar electrical conductivity as the human body [1], which demonstrated that the conductivity change interface can be clearly detected. In their experiments, a plane probe was used as an ultrasonic excitation source. However, the cross-sectional area of the ultrasound beam was difficult to manufacture within a small area while maintaining a high stimulating power, and the average sound intensity in the ultrasonic propagation path was often weak. The resolution of that method could be further improved. In current MAET research, either a plane probe [6] or a high-voltage narrow pulse excitation signal was used, which resulted in the poor resolution of the conductivity imaging and the excessive instantaneous excitation power of the probe. To solve these problems, we designed and implemented a MAET system using a high-power focal probe and a chirp signal with sweep frequency ranging from 2 MHz to 3 MHz. Based on the MAET system, we adopted the linearly frequency-modulated method to reduce the peak stimulating power. Both sfMAET-CPS (single-focus B-scan imaging) and the mfMAET-CPS (multifocus imaging) can be used for detecting the interfaces of conductivity variations of homogeneous phantoms and the accuracy and feasibility of the mfMAET-CPS was verified by experiments. The detailed technical comparisons between our proposed system and the previous MAET methods are shown in Table 1.

Ultrasonic focus excitations at each focal point and nearby are relatively powerful and uniform. Therefore, the amplitude of the particle vibration near the focal point is large, and the conductivity amplitude near the focal point can be detected with high amplitude. Considering the advantages of exacting the conductivity value from the points near the focal point, and combined with the advantages of the large vibration amplitude at the focal point and the linear frequency modulation method, we conducted in-depth research on the mfMAET-CPS using a stepscan focus excitation method in the *z*-axis. By contrast with previous setups and methods, the stepscan focus excitation method and the linearly frequency-modulated without inverse process were applied to our experiments, a high-power focal probe was applied to generate an ultrasonic pulse to make iron vibrate in an imaging tissue, and a pair of electrodes was used to collect the MAET signal. In addition, the relevant accuracy and comparative experiments were carried out on a low-conductivity gelatin phantom with a cuboid-shaped hole in the middle to illustrate the performance of the mfMAET-CPS, and the measured thicknesses of the phantom showed good agreement with the thickness of the phantom. The reconstructed 2D conductivity distribution with the mfMAET-CPS method can be comparable to that obtained through Lorentz force electrical impedance tomography (LFEIT) method by [20], and could obtain the similar performance of detecting electrical conductivity variations as the MAET using high voltage narrow pulse.

## 2. Theory of Magneto-Acoustic-Electrical Tomography with Chirp Pulse Stimulation (MAET-CPS)

In our MAET-CPS detection system, to cut down the peak power requirement of the stimulating probe while maintaining the same average power, we conducted in-depth research of MAET using a linear frequency modulation pulse to reduce the peak exciting power to the ultrasound probe, and a chirp pulse with the frequency sweeping linear from 2 MHz to 3 MHz, the amplitude of 200-mV and the chirp pulse duration of 1000 μs was generated by a signal generator and sent to a two-output power splitter. A one-way output signal was sent directly to the Verasonics system, and another output signal was sent to the ultrasonic transmitting probe after power amplification. The stimulating chirp signal to the ultrasound transducer can be denoted as Equation (1), with the frequency sweeping linearly from $f_{0}$ to $f_{0}+∆f$.

(1) $$T_{r}=A_{1}\sin\left\{2\pi \left[f_{0}+\frac{∆f}{2T}\left(t-kT_{n}\right)\right]t+\phi_{1}\right\};kT_{n}\leq t\leq kT_{n}+T$$

where $A_{1}$ is the amplitude of the stimulating signal, ∆f is the bandwidth of the modulation frequency, $f_{0}$ is the initial frequency, T is the pulse duration (also called the frequency modulation period), $T_{r}$ is the stimulating signal, $T_{n}$ is the chirp-pulse repeat period, $\phi_{1}$ is the initial phase, and k is an integer, *k* = 0, 1, ···, *N* − 1; 0 ≤ *t* ≤ *T*.

After power amplification, the chirp pulse was used for stimulating the ultrasound transducer to produce the transmitted ultrasound pressure in the target sample. As the frequency of the ultrasound stimulating pulse increased linearly with time, the amplitude of the received signal also changed with time, and the integral of the ultrasound pressure in the target sample led to amplitude variation of the receiving signal, which brought a new frequency component into the received signal. The received signal had a time delay compared with the stimulating signal and, therefore, the receiving signal can be described as Equation (2):

(2) $$R_{e}\;=\;A(t)\sin\{2\pi \left(f_{0}+\frac{∆f}{2T}\left(t-kT_{n}-\frac{R}{c}\right)\right)\left(t-\frac{R}{c}\right)+\phi_{2}\};kT_{n}+\frac{R}{c}\leq t\leq kT_{n}+T+\frac{R}{c}$$

where A(t) is the amplitude of the received signal, *c* is the ultrasound propagation velocity in the tissue, *R* is the distance of the conductivity variation to the ultrasound probe, $R_{e}$ is the received signal, and $\phi_{2}$ is the initial phase.

(3) $$\begin{array}{l} R_{e}\;\cdot \;T_{r}\;=\;\frac{A_{1}A(t)}{2}\cos\left\{\frac{2\pi ∆f}{T}\cdot (t-kT_{n})\cdot \frac{R}{c}+2\pi (f_{0}-\frac{∆f}{2T}\cdot \frac{R}{c})\cdot \frac{R}{c}+\phi_{2}-\phi_{1}\right\}\\ -\frac{A_{1}A(t)}{2}\cos\left\{4\pi \left[f_{0}+\frac{∆f}{2T}\left(t-kT_{n}-\frac{R}{c}\right)\left(t-kT_{n}\right)-2\pi (f_{0}-\frac{∆f}{2T}\cdot \frac{R}{c})\cdot \frac{R}{c}+\phi_{2}+\phi_{1}\right]\right\} \end{array}$$

In Equation (3), the multiplication of the stimulating signal and receiving signal could be deduced and processed by a low-pass filter (*LPF*) with a 0.6-MHz cut-off frequency. The amplitude of the intermediate frequency (IF) signal is proportional to the time of flight of the compression waves from the excitation transducer to the target sample, which can be written as Equation (4):

(4) $$LPF\left\{R_{e}\;\cdot \;T_{r}\right\}\;\propto \;\cos[2\pi \left(t-kT_{n}\right)\frac{∆f}{T}\cdot \frac{R}{c}+2\pi \left(f_{0}-\frac{∆f}{T}\cdot \frac{R}{c}\right)\frac{R}{c}+\phi_{2}-\phi_{1}]$$

**Figure 1:**
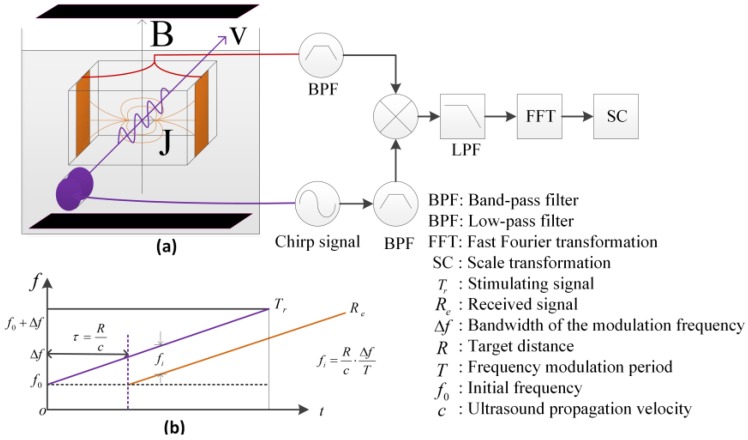
Magneto-acoustic-electrical tomography (MAET) imaging process using linearly frequency-modulated theory (MAET-chirp pulse stimulation (CPS)): (**a**) principle of the MAET-CPS; (**b**) frequency characteristics of the stimulating signal and the received signal.

As shown in Equation (4), the frequency difference (the intermediate frequency) between the instantaneous stimulating signal and receiving signal can be expressed as Equation (5):

(5) $$f_{i}\;=\;\frac{∆f}{T}\cdot \frac{R}{c}$$

where $f_{i}$ is the IF signal and is linearly related to the stimulating depth of the electrical conductivity variation. After the fast Fourier transformation (FFT) and the scale transformation, the interface position of the conductivity variation can be reconstructed from the spectrum of the IF signal. Then, the electrical conductivity curves along the ultrasound stimulating direction can be derived, and the reconstructed B-scan imaging of the conductivity distribution within the target sample can be obtained after combining the electrical conductivity curves with the motion positions of the probe; the imaging principle of the MAET-CPS is shown in Figure 1a and the frequency characteristics of the stimulating signal and the received signal are shown in Figure 1b.

## 3. Experimental Setup and Methods

### 3.1. System Design

The proposed MAET-CPS system consisted of an ultrasonic focusing probe, a three-dimensional motion system, a Verasonics acquisition system, two signal generators, a C-shaped static magnet, a power amplifier, two impedance matchers, a power splitter, a high power single element transducer, two silver-plated copper electrodes, and a plastic sink container. A 3D motion system (MC600, ZOLIX Instruments Inc., Beijing, China) was used to control the movement of the probe and included a motion controller and a motion platform. A focusing probe (CDC-10963-4, IMASONIC Inc., Voray sur l’Ognon, France) was used to generate the ultrasonic pulse to make local ultrasonic vibration on the ultrasonic path. The Verasonics acquisition system (Vantage Advantage 256, Kirkland, USA) was adopted to perform digital signal processing of the received voltage signal and the stimulating signal, such as preamplification, 14-bit analog-to-digital conversion, sampling, amplifying, band-pass filtering, digital multiplication, low-pass filtering, fast Fourier transformation (FFT), and scale transformation. The MC600 motion controller (MC600, ZOLIX Instruments Inc., Beijing, China) was used for system timing sequential control and performing the step scanning motion. Signal generator 2 (AFG3102, Tektronics Inc., Rocky Point, NY, USA) was used to generate a beam of linear frequency modulation excitation signals with a bandwidth of 1 MHz, a center frequency of 2.5 MHz, and a linear frequency modulation period of 1 ms. To improve the signal to noise ratio of the MAET-CPS system, Signal generator 1 (AFG3102, Tektronics Inc., USA) was used to generate 10 rectangular pulse signals with the period of 1 s for signal averaging. The system’s physical diagram is shown in Figure 1a.

**Figure 2:**
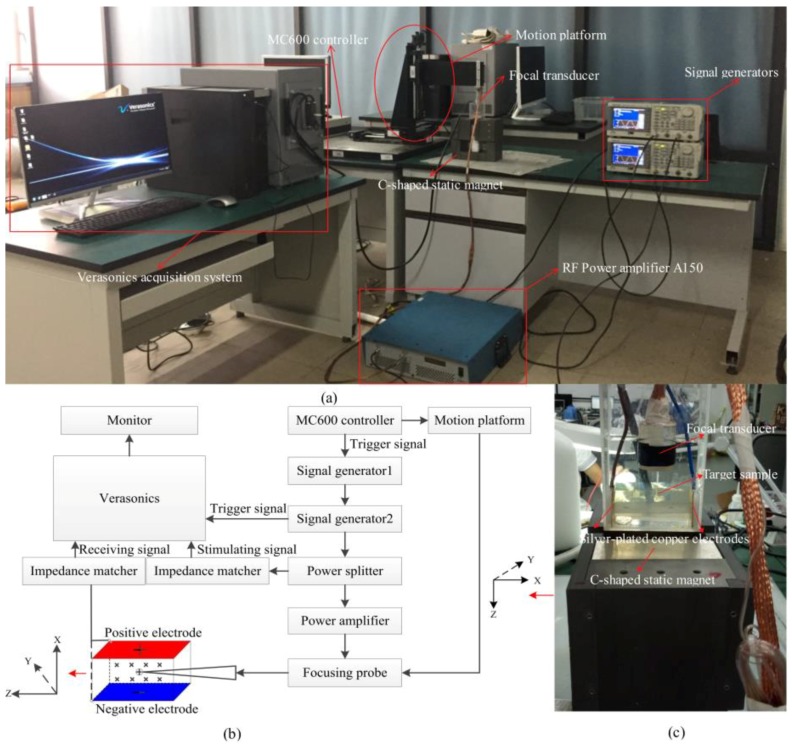
System composition of the MAET-CPS detection system: (**a**) physical diagram; (**b**) connection diagram; and (**c**) the detection front end.

In our MAET system, the MC600 motion controller generated a trigger signal to switch on or off the signal generator 1, and the signal generator 2 was triggered by the signal generator 1. Each trigger signal produced by the signal generator 1 generated a 10-time impulse signal to the signal generator 2 to turn on or off the output of the excitation signal. After generating a change of the level signal at each time trigger, a beam of chirp pulse signals was produced by the signal generator 2 and separated into two ways after a two-output power splitter. A one-way output signal was then received by the Verasonics system through a double-shielded cable and an impedance matcher, and the other output signal was sent to a high-power focusing transducer after a 53 dB power amplification (RF Power Amplifier A150, Electronics & innovation Inc., New York, NY, USA). Furthermore, a pair of silver-plated copper electrodes was closely attached to both sides of the phantom and was connected to the Verasonics system after the impedance matcher. Subsequently, the imaging sample was placed and fixed in a plastic sink container, which was placed in the center of a C-shaped static magnetic field of 100 × 90 × 40 mm, which was generated by two cube Nd-Fe-B magnets (100 × 100 × 40 mm), and the magnetic field within the central cube of 64 cm^3^ between the two magnets was approximately 0.45 T. In addition, the probe was fixed on the motion platform, and then deionized water was poured into the tank. After the motion platform was calibrated, motion control commands from the MC600 motion controller were sent to the motion platform to control the movement of the probe. When the probe moved to the target position, the MC600 motion controller sent a trigger signal to turn on the signal generator 2 to generate a beam of chirp signals to activate the ultrasonic focusing transducer. Meanwhile, the excitation signal and the received surface voltage of the sample were detected by the Verasonics system and the conductivity curve in the *z*-axis was obtained after merging the electrode detection signal and the ultrasonic excitation signal. By moving the probe along the *x*-axis to the next position and repeating the aforementioned operation, the electrical conductivity distribution in the xz plane area was obtained by merging the electrical conductivity curves along the ultrasonic propagation direction with the corresponding position information in the *x*-axis. The connection diagram of our MAET system is shown in Figure 2b and the detection front end is shown in Figure 2c.

### 3.2. Single-Focus B-Scan Imaging MAET-CPS (sfMAET-CPS) and Multifocus Imaging MAET (mfMAET) Algorithms

**Figure 3:**
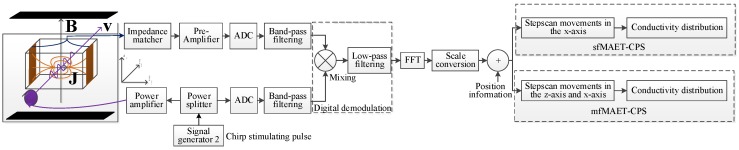
Algorithm flow chart of the single-focus B-scan imaging MAET with chirp pulse stimulation (sfMAET-CPS) and the multifocus imaging MAET with CPS (mfMAET-CPS).

In our proposed MAET system, MATLAB was utilized as the programming language, and data acquisition and movement of the probe were controlled by the MC600 controller. A pair of silver-plated copper electrodes attached to the surface of the imaging body was used to detect the surface voltage signal of the phantom. In addition, both the chirp excitation signal and the received voltage signal were used to perform the preamplification, the ADC acquisition, the mean calculation, and the band-pass filtering through the Verasonics acquisition system. By mixing the signals, the intermediate frequency (IF) signal was derived after low-pass filtering and fast Fourier transformation (FFT), and the conductivity curve along the pulse propagation direction was achieved after scale transformation (SC). According to the different stepscan movements, and combined with the position information of the stimulating probe and the conductivity curve, the electrical conductivity distributions of the sfMAET-CPS and the mfMAET-CPS were derived by two different reconstruction algorithms, respectively. The process flowchart of the two methods is presented in Figure 3.

### 3.3. Phantom Design

To verify the focal point effect that the amplitudes of the conductivity variation varied with the different single-point focal positions, pig-skin powder, cellulose, NaCl, and water were chosen to make homogeneous phantoms with conductivities similar to that of the biological tissue. A homogenous gelatin phantom (75 × 46 × 30 mm) with 0.5% NaCl and a cuboid-shaped hole of 12 × 10 × 30 mm in the middle and a same homogenous phantom without a hole were exploited as the imaging target samples for demonstrating the effect. To test the feasibility and the accuracy of the mfMAET-CPS method, the above homogenous phantom with a hole was used to conduct performance tests, the mean value obtained by the ultrasound B-mode and the calculated values obtained by the mfMAET-CPS were compared in our study, and a homogenous gelatin phantom (75 × 29 × 30 mm) with 0.5% NaCl and a cuboid-shaped hole of 10 × 8 × 30 mm in the middle was conducted for the imaging experiments of the 2D conductivity distribution. In our setup, both the imaging sample and electrodes were immersed in water, and the front end of the ultrasound probe was dipped into water for good acoustic coupling.

## 4. Effect of Focusing Probe on Conductivity Imaging

**Figure 4:**
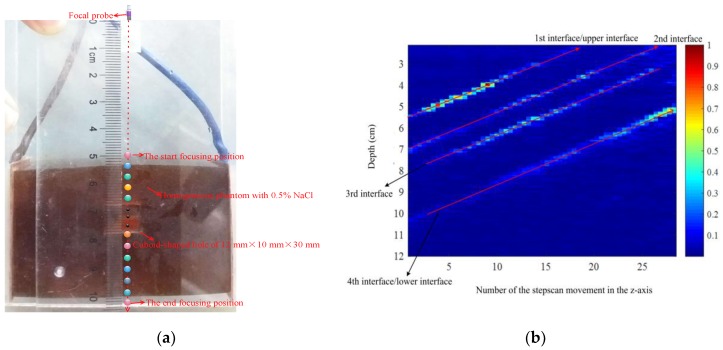
The influence of single focus on the experiment: (**a**) the movement track of the focal point position, which passed through a cuboid-shaped hole; and (**b**) the brightness curves with the interface information.

To test the effect of the different single-point focal positions on the amplitude of electrical conductivity, a focal probe was controlled for step scanning only in the *z*-axis. The phantom with a cuboid-shaped hole was exploited to conduct stepscan movements for demonstrating the effect of single focal-point excitation at different positions on conductivity imaging, and an ultrasonic single-element probe with a depth of focus of 50 ± 2 mm was placed above the hole and placed at the 54 mm position above the upper surface of the homogenous phantom. When the focal point was at the start focusing position, the probe sent 10 chirp signals to stimulate the target phantom; 10 surface voltages of the phantom were received by a pair of silver-plated copper electrodes placed close to both sides of the target sample. Both stimulating signals and received signals were averaged and a conductivity curve from the probe to the bottom of the phantom was obtained after the digital coherent demodulation between the instantaneous stimulating signal and the received signal. Then, the probe moved to a step length (1.8 mm) to reach the second focal point to obtain the second conductivity curve along the ultrasonic excitation direction, repeating the aforementioned operation and stepped excitation of the phantom. Thirty stepscan movements of the probe were conducted in the *z*-axis and the focal point of the probe passed through the hole and moved from the start focusing position to the end focusing position. The movement track of the stimulating position at each focal point is shown in Figure 4a, in which the influence of single-focus imaging on the amplitudes of conductivity variation was clearly presented. When the focus point was close to the first interface, the conductivity amplitude at the first interface position became increasingly brighter. As the focal point was farther away from the first interface, the electrical conductivity amplitude at the first interface position became increasingly weaker. When the focal point was close to the second, third and fourth interfaces, the conductivity amplitude of the probe was also getting increasingly brighter and then became darker as the focal point was farther away from the interfaces. As the focal point of the probe was fixed from the probe position, when the probe stepped up excitation of the phantom, the conductivity curves obtained at each excitation position were from the probe to the bottom of the phantom, so the obtained conductivity curves referred to the position of the probe. After normalization of the conductivity values extracted from the 30 conductivity curves, the conductivity values were displayed by the color brightness. The above results could be reflected by the brightness of the conductivity of the four interfaces as shown in Figure 4b, and the unit of measure of the brightness was the conductivity amplitude after normalization.

**Figure 5:**
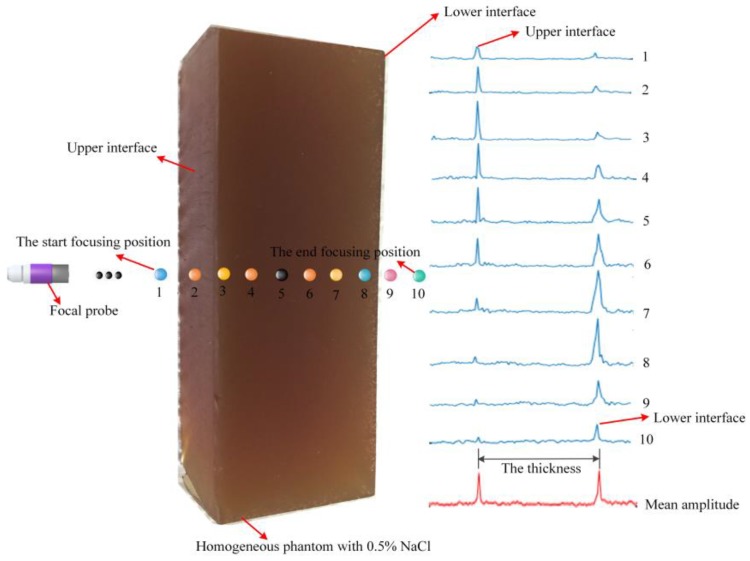
The movement track of the focal point position and the amplitude variation of conductivity curves of 10 step-scan movements in the depth direction.

To further verify the influence of the different single-point focal positions on the amplitudes of conductivity variation, a homogenous gelatin phantom was used to carry out an experiment. In the beginning, the probe was placed at the 54 mm position above the upper surface of the homogenous phantom, and the distance from the start focusing position to the upper interface of the phantom was approximately 4 mm. Then, the chirp stimulating signal was generated and the surface voltage signal was received by the electrodes. When the focal point was at the start focusing position, the conductivity curve along the ultrasound stimulation direction was obtained after processing by the Verasonics system, and then the focal point moved down a step length to the second focusing position to get the second conductivity curve, repeating the aforementioned operation. The focal point of the probe was controlled to move from the start focusing position to the end focusing position, and 10 stepscan movements of the probe were carried out in the *z*-axis. After combining the ultrasonic excitation signal and the measured voltage signal from the surface of the tissue, the electrical conductivity curves were derived when focus points were at different single-point excitation positions, and the amplitude variations of the conductivity curve of the 10 stepscan movements in the *z*-axis are shown in Figure 5. From Figure 5, the conductivity amplitudes at the two interfaces were seriously influenced by the excitation positions of the single focus point, The amplitude of the conductivity curve at the upper interface became larger and then gradually became smaller as the single-focus point moved, and the amplitude of the conductivity curve at the lower interface gradually increased when the focal point moved gradually closer to the lower interface, which then gradually decreased as the focal point passed through the lower interface. In addition, the average of 10 conductivity amplitudes at the upper interface was almost equal to that at the lower interface; the two peak values on each conductivity curve represented the conductivity variations of the two interfaces of the phantom, and the measured distance between the two interfaces was close to the thickness of the phantom. To sum up, the above phenomena clearly illustrated the relationship between the conductivity variation at the two interfaces of the phantom and the different single-point focal positions, which is shown in Figure 4b and Figure 5.

## 5. MAET Imaging Methods

### 5.1. The sfMAET-CPS Imaging Method

**Figure 6:**
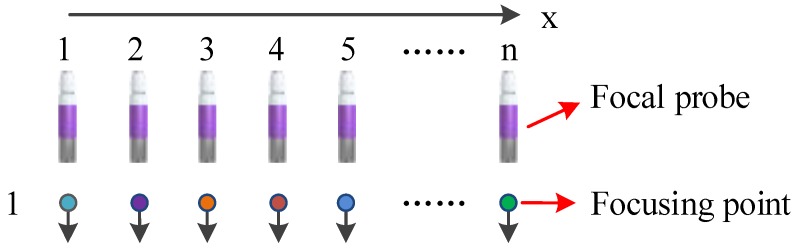
The imaging process of the sfMAET-CPS.

In the sfMAET-CPS, a focal probe was used for stimulating a target sample along the *z*-axis, and the step motions of the probe along the *x*-axis were controlled by the MC600 controller. In the imaging processing, first, the probe was adjusted in a start stimulating position. Then, the probe sent 10 chirp signals to stimulate the target phantom; 10 surface voltages of the phantom were received by a pair of silver-plated copper electrodes placed close to both sides of the target sample; and after digital coherent demodulation, a conductivity curve along the *z*-axis was obtained. Second, the probe was moved along the *x*-axis for a step length, and then, the second conductivity curve along the *z*-axis was derived by repeating the above mentioned process and different conductivity curves along the *z*-axis were achieved. After finishing the n stepping motions in the *x*-axis, the 2D conductivity contribution in the xz plane area was presented after combining n conductivity curves with the probe position in the *x*-axis. The imaging process of the sfMAET-CPS is shown in Figure 6.

Since the sfMAET-CPS imaging was extremely affected by the position of the focal point, the focal point within the imaging sample was hard to accurately estimate, and the detected conductivity amplitude was susceptible to a focus point position, it was difficult to guarantee that the conductivity amplitudes measured at different excitation depth were the same. In order to overcome the influence of the focal point on the conductivity imaging and utilize the advantages of the focal probe at the focal point and nearby, we proposed the mfMAET-CPS method.

### 5.2. The Focusing Imaging Method (mfMAET-CPS)

**Figure 7:**
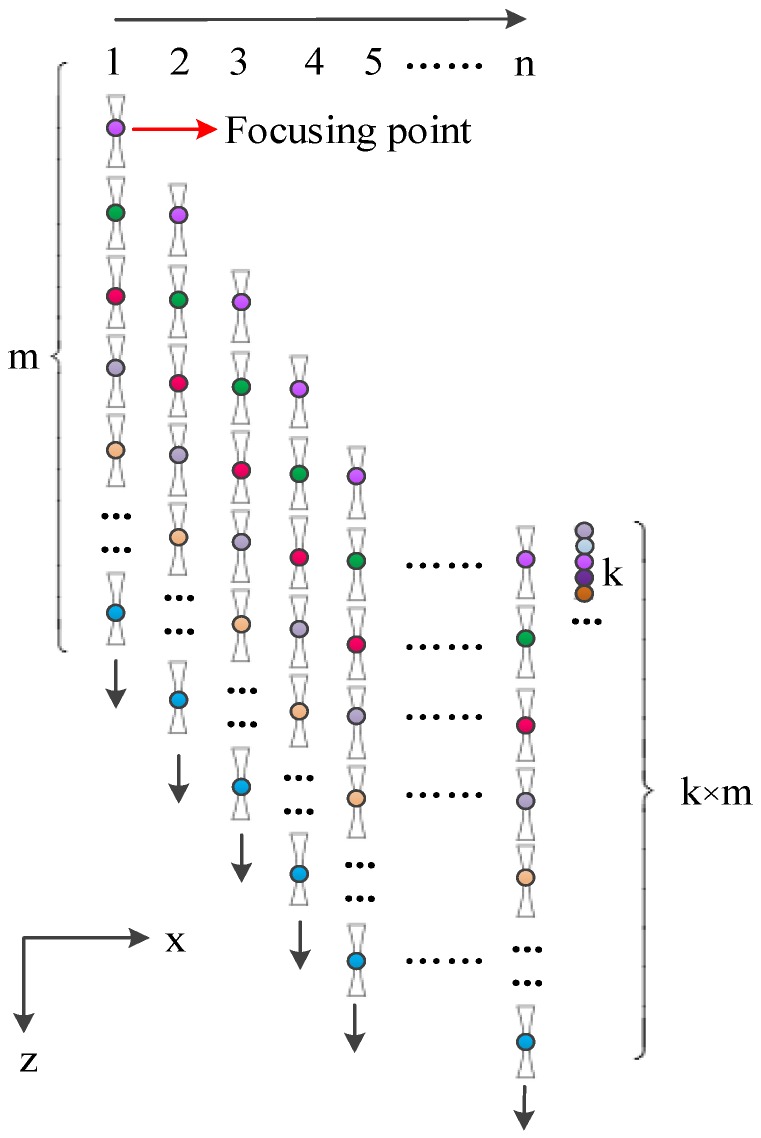
The imaging process of mfMAET-CPS.

The vibration amplitudes of the particles generated in the imaging body were almost the same when the same focal probe stimulated a phantom at the different focal point positions. Thus, the obtained electrical conductivity amplitudes near the focal point positions were probably the same when the conductivity variations were similar. Based on this principle, a new conductivity imaging modality known as the focusing imaging method (mfMAET-CPS) was introduced in this work. Similar to the sfMAET-CPS method, a focal probe was used for step scanning along *z*-axis and *x*-axis and was controlled by the MC600 controller. In order to improve the conductivity resolution and reduce the imaging time, more conductivity values near the focal point were exploited for increasing the imaging points. In the mfMAET-CPS, the focal probe was adjusted at the start focusing position of the imaging area, then, the probe sent 10 chirp signals to stimulate the target phantom; 10 surface voltages of the phantom were received by a pair of silver-plated copper electrodes placed close to both sides of the target sample. Both the stimulating signals and the received signals were averaged and a conductivity curve from the probe to the bottom of the phantom was obtained after the digital coherent demodulation of the two signals, and k conductivity values at the focal point and its adjacent points were extracted from the conductivity curve, and then the focal probe was moved down a step length to the next excitation position in the *z*-axis. Total m conductivity curves along the ultrasonic propagation direction were derived from the ultrasonic stimulating signals and the received signals. Therefore, there were m × k conductivity amplitudes at each focal point and nearby points, which were extracted from the m conductivity curves. With those data and their position information, a synthetic conductivity curve along the ultrasonic propagation direction was derived. Subsequently, the probe went back to the initial position (the start focusing position) and then moved forward a unit in the *x*-axis, repeating the aforementioned process, and then other m × k amplitudes at each focal point and nearby points along the *z*-axis were achieved. After finishing the n stepscan movements in the x direction, n synthetic conductivity curves with m × *n* × k conductivity values in the depth direction were achieved and the 2D conductivity contribution in the xz plane area was achieved after combining the scanning position in the *x*-axis with the *n* synthetic conductivity curves (total k × m × *n* conductivity amplitudes at each focal point and its nearby points). The imaging process diagram of the mfMAET-CPS is shown in Figure 7.

**Figure 8:**
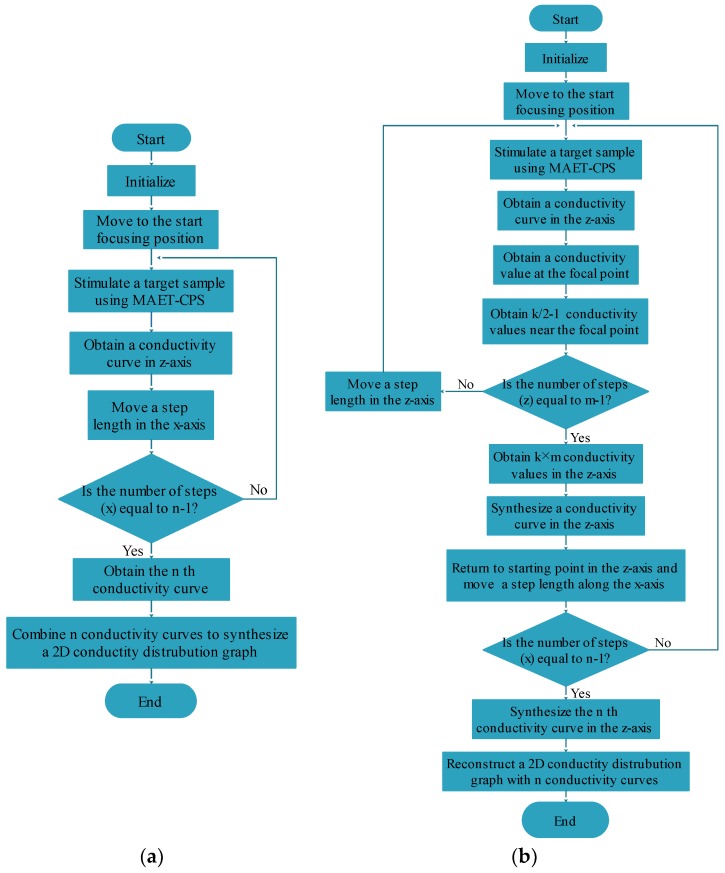
The motion control and image synthesis processes: (**a**) the sfMAET-CPS; and (**b**) the mfMAET-CPS.

In our reaserach, a digital coherent demodulation algorithm, a step motion control algorithm and an image synthesis algorithm were used in the sfMAET-CPS and mfMAET-CPS imaging, respectively. No overlapping measuring points were used among the two methods, and the motion control and image synthesis processes of the two imaging methods were denoted as Figure 8.

## 6. Results and Discussion

### 6.1. Accuracy Measurement of mfMAET-CPS

**Figure 9:**
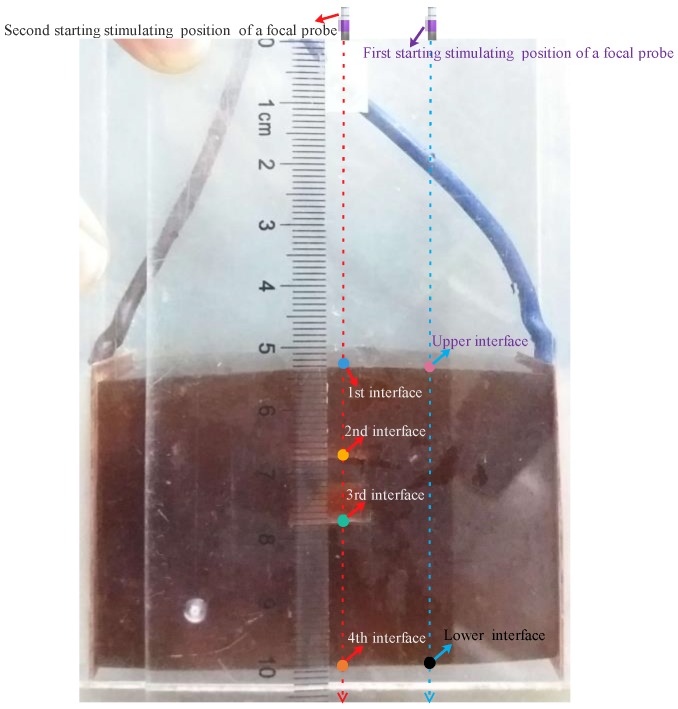
The stimulating positions of two experiments.

**Figure 10:**
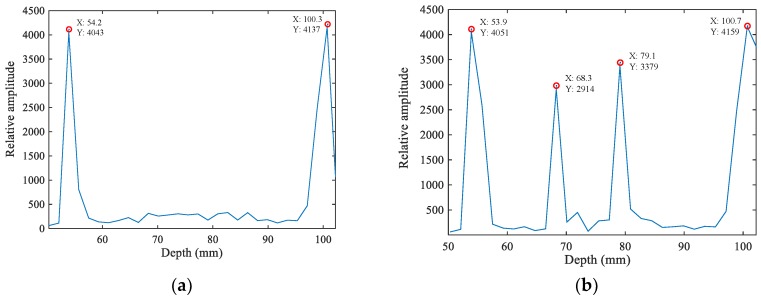
Results of accuracy measurement of mfMAET-CPS: (**a**) two interfaces detected by mfMAET-CPS; and (**b**) four interfaces detected by mfMAET-CPS.

In order to test the accuracy of the mfMAET-CPS, a homogenous gelatin phantom with a cuboid-shaped hole of 12 × 10 × 30 mm in the middle was used as the target sample. An ultrasonic focal probe was placed at the 54 mm position above the upper surface of the phantom, and the stimulating positions of the two experiments are shown in Figure 9. When the probe was placed at the first starting stimulating position (i.e., ultrasound passed through only two layers of conductivity change interface), the probe sent 10 chirp signals to stimulate the target phantom; 10 surface voltages of the phantom were received by a pair of silver-plated copper electrodes .Then, a conductivity curve was formed and conductivity amplitude at focal point was obtained. A total of 30 stepscan movements along the ultrasonic propagation direction were performed by repeating the aforementioned operations, and then a synthetic electrical conductivity curve using 30 conductivity curves obtained at different excitation depths was derived by the mfMAET-CPS, which is shown in Figure 10a. Two peak points with the same levels of amplitude—(54.2, 4043) and (100.3, 4137)—were also achieved and the two peak points meant the maximum peak points of conductivity variation. In additon, the two peak values stood for the positions of condutivity variation in the *z*-axis. By measuring the distance between the two peak points, the calculated distance between the two interfaces of conductivity variation was 46.1 mm. In additon, the ultrasound B-mode imaging method was used to image the target phantom by our Verasonics system, five position points of each interface of the B-mode imaging were randomly exacted and averaged, and the mean value was used as the measured value; therefore, the calculated thickness between the upper and lower interface of the target sample was obtained, and the calculated values obtained by the mfMAET-CPS were close to the measured phantom thickness of 46.6 mm. The detailed interface positions were measured and are presented in Table 2. The accuracy in the *z*-axis was as high as 98%, which validated the accuracy of the mfMAET-CPS and indicated that the MAET-CPS can be applied to detect the interface of conductivity variation of the phantom.

In order to further test the accuracy of the mfMAET-CPS, the ultrasound B-mode imaging method was used to image the target phantom by our Verasonics system, the focal point of the probe was moved to the second starting stimulating position (the ultrasound passed through four layers of the conductivity change interface and the probe was placed directly above the hole at the 54-mm position above the first interface of the phantom), and the stepscan movements were repeated in order to obtain the synthetic conductivity curve. As shown in Figure 10b, the four conductivity peak points ((53.9, 4051), (68.3, 2914), (79.1, 3379), (100.7, 4159)) were obtained, and the amplitudes of the first peak and the fourth peak were similar. The calculated distance between them was 46.8 mm, which was consistent with the measured thickness of 46.9 mm. Meanwhile, the detected conductivity amplitudes between the second peak and the third peak were similar, and the measured distance among them was 10.7 mm, which was close to the measured thickness of 10.8 mm. The detailed interface positions calculated by the mfMAET-CPS and measured by ultrasonic B-mode imaging are shown in Table 3. The results demonstrated that the accuracy of mfMAET-CPS was high and the theory of mfMAET-CPS was correct and feasible, which clearly indicated that the mfMAET-CPS could be used for detecting boundary regions of conductivity variation.

### 6.2. Comparison of the Two Different Methods

**Figure 11:**
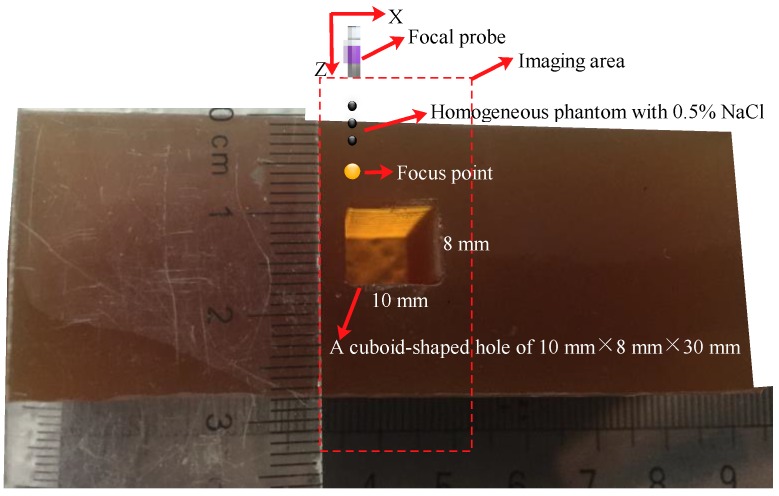
The uniform phantom for testing.

**Figure 12:**
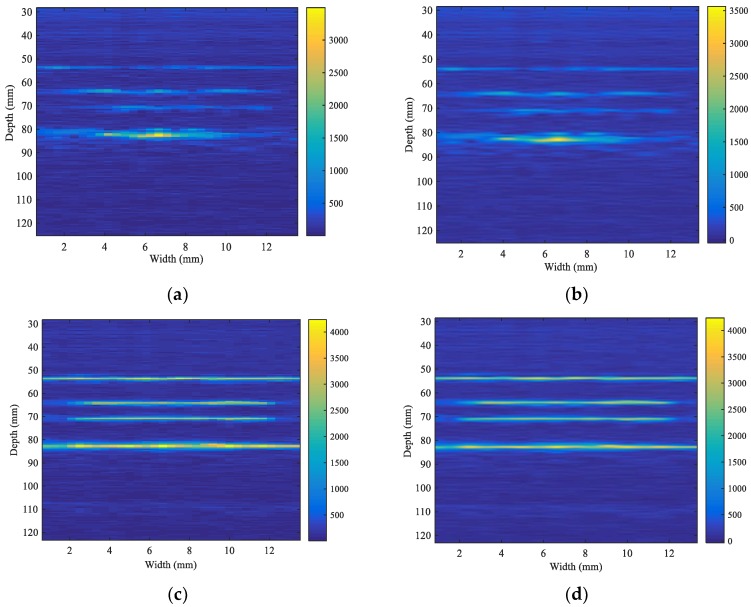
Imaging results: (**a**) conductivity distribution of the sfMAET-CPS; (**b**) conductivity distribution of the sfMAET-CPS after being processed by the linear interpolation algorithm; (**c**) conductivity distribution of the mfMAET-CPS; and (**d**) conductivity distribution of the mfMAET-CPS after being processed by the linear interpolation algorithm.

A homogenous gelatin phantom (75 × 29 × 30 mm) with a cuboid-shaped hole of 10 × 8 × 30 mm in the middle was conducted for conductivity imaging experiments. As shown in Figure 11, an ultrasonic focal probe was placed above the upper surface of the phantom and was used for conductivity imaging, and 2D electrical conductivity distributions in the imaging area were clearly obtained by the single-focus B-scan imaging and the mfMAET-CPS, respectively, which are shown in Figure 12a,c. In two different imaging experiments, the stepscan movement in the *x*-axis was set as 0.45 mm, the stepping motion along the *x*-axis was performed 31 times, and the mfMAET-CPS was performed 30 times along the *z*-axis and each step length was set to 1.8 mm. The conductivity imaging results are shown in Figure 12, in which Figure 12a is the 2D conductivity distribution of the sfMAET-CPS, Figure 12b is the 2D conductivity distribution of the sfMAET-CPS after being processed by the linear interpolation algorithm, Figure 12c shows the 2D conductivity distribution of the mfMAET-CPS, and Figure 12d is the 2D conductivity distribution of the mfMAET-CPS after being processed by a linear interpolation algorithm.

As shown in Figure 12a,c, both the sfMAET-CPS and the mfMAET-CPS can be used for distinguishing the four interfaces of the target phantom, but the imaging resolution for the sfMAET-CPS imaging still needed to further improve due to the derived conductivity distribution being extremely susceptible to the focal point position. Compared with the sfMAET-CPS imaging, the imaging effect of the mfMAET-CPS was better because the vibration amplitudes at each focal point and its nearby points were probably the same, and for the mfMAET-CPS, the conductivity value was similar at the area that had the same conductivity change. Unlike the sfMAET-CPS, the mfMAET-CPS adopted the multifocus point values for electrical conductivity imaging, and the electrical conductivity values were not only exacted at each focal point but also at its nearby points in the *z*-axis. Thus, the mfMAET-CPS overcame the disadvantage that the sound intensity distribution of the focal probe was inhomogeneous. To better show the interface positions of conductivity variation, the linear interpolation algorithm was only used for smoothing the 2D conductivity distributions of the sfMAET-CPS and the mfMAET-CPS, and the imaging effects obtained are shown in Figure 12b,d, respectively. Compared to the original image, the linear interpolation algorithm improved the imaging smoothness. The interface positions of conductivity variations measured by the msMAET-CPS were highly consistent with those of the ultrasonic B-mode scanning image, thereby further demonstrating the accuracy of our msMAET-CPS method.

## 7. Conclusions

In our proposed detection system, the sfMAET-CPS and mfMAET-CPS were used for conductivity measurement. Accuracy experiments of the mfMAET-CPS and contrasting experiments were performed on the same homogeneous phantom, and 2D conductivity distributions were clearly obtained by the two imaging methods, from which we drew the following conclusions:

(1) The sfMAET-CPS imaging can be used for dectecting the interface positions of conductivity variations, but it was easily affected by the position of the focal point, which presented low imaging resolution.
(2) The mfMAET-CPS took advantage of the maximum vibration amplitude at the focal point, and the positions of each focal point and its nearby points had good sound intensity uniformity. Only conductivity values at the focal points and nearby points were extracted, which led to a better imaging effect than the sfMAET-CPS imaging. However, due to the limited experimental conditions, the conductivity values measured at the focal point and nearby points were limited by the step length of each stepscan movement, which resulted in a limited resolution. However, in future, by means of optimizing the system algorithm and changing the scanning method, such as electronic scanning instead of mechanical scanning, the imaging resolution is expected to be significantly improved by reducing the scanning steps.
(3) In addition, a linear interpolation algorithm was used to process the conductivity distribution obtained by the sfMAET-CPS and the mfMAET-CPS. It was proved that a 2D linear interpolation algorithm increased the smoothness of conductivity imaging and better showed the interface positions of conductivity variation.

In this paper, in contrast to previous setups, a focal power probe was adopted for a vibration excitation source, and a chirp excitation signal was used as an excitation signal in our proposed detection system. The influence of the different single-point focal positions on the amplitudes of conductivity variation was presented for the first time, a novel imaging method to improve axial resolution of conductivity imaging was proposed, and the feasibility and accuracy of the mfMAET-CPSmethod were verified by the relevant experiments. Finally, the imaging effects of the mfMAET-CPS and the mfMAET-CPS were compared by experiment.

## Figures and Tables

**Table 1:** Technical comparisons between our proposed system and other similar approaches.

Item	Probe Type	Excitation Source Signal	Imaging Method
Magneto-acoustic-electrical tomography (MAET) [1]	Plane probe	High-voltage narrow pulse	Reciprocity theorem
LFEIT [20]	Plane probe	1.4–3.4 M Hz chirp signal	Linearly frequency-modulated
Single-focus B-scan imaging MAET with chirp pulse stimulation (sfMAET-CPS)	Focal probe	2–3 M Hz chirp signal	Linearly frequency-modulated
Multifocus imaging MAET with chirp pulse stimulation (mfMAET-CPS)	Focal probe	2–3 M Hz chirp signal	Linearly frequency-modulated

**Table 2:** Accuracy analysis of two interfaces by mfMAET-CPS.

Items	Upper Interface	Lower Interface	Thickness
Calculated value(mm)	54.2	100.3	46.1
Measured value (mm)	53.6	100.2	46.6
Accuracy (%)	98.8	99.8	98.9

**Table 3:** Accuracy analysis of four interfaces by mfMFMAET-CPS.

Items	1st Interface	2nd Interface	3rd Interface	4th Interface	Gap 1–2	Gap 2–3	Gap 3–4
Calculated value (mm)	53.9	68.3	79.1	100.7	14.4	10.7	21.7
Measured value (mm)	53.6	67.7	78.5	100.5	14.1	10.8	22.0
Accuracy (%)	99.4	99.1	99.2	99.8	97.9	99.1	98.6

## References

[1] Li Y.Y., Liu G.Q., Xia H., Xia Z.W. (2017). Numerical Simulations and Experimental Study of Magneto-Acousto-Electrical Tomography With Plane Transducer. IEEE Trans. Magn..

[2] Liu G.Q., Huang X., Xia H., Wu S.Z. (2013). Magnetoacoustic tomography with current injection. Sci. Bull..

[3] Guo L., Liu G.F., Yang Y.J., Liu G.Q. (2015). Vector based reconstruction method in magneto-acousto-electrical tomography with magnetic induction. Chin. Phys. Lett..

[4] Lv J., Liu G., Wang X., Xia H. (2016). A method of the forward problem for magneto-acousto-electrical tomography. Technol. Health Care.

[5] Blott B.H., Daniell G.J., Meeson S. (1998). Nonlinear reconstruction constrained by image properties in electrical impedance tomography. Phys. Med. Biol..

[6] Sun Z.S., Liu G.Q., Xia H. (2017). Lorentz force electrical impedance tomography using pulse compression technique. Chin. Phys. B.

[7] Kunyansky L., Ingram C.P., Witte R.S. (2017). Rotational magneto-acousto-electric tomography (MAET): Theory and experimental validation. Phys. Med. Biol..

[8] Munish C., Chul J.W., Joong K.H., In K.O., Je W.E. (2013). Optimization of magnetic flux density for fast MREIT conductivity imaging using multi-echo interleaved partial fourier acquisitions. Biomed. Eng. Online.

[9] Haider S., Hrbek A., Xu Y. (2008). Magneto-acousto-electrical tomography: A potential method for imaging current density and electrical impedance. Physiol. Meas..

[10] Sun Z.T., Liu G.Q., Guo L., Xia H., Wang X. (2016). Effect of the secondary process on mass point vibration velocity propagation in magneto-acoustic tomography and magneto-acousto-electrical tomography. Technol. Health Care Off. J. Eur. Soc. Eng. Med..

[11] Xu Y., He B. (2010). Magnetoacoustic tomography with magnetic induction (MAT-MI). IEEE Trans. Med. Imaging.

[12] Li X., He B. Magnetoacoustic tomography with magnetic induction (MAT-MI) for electrical conductivity imaging. Proceedings of the 2009 Annual International Conference of the IEEE Engineering in Medicine and Biology Society.

[13] Guo L., Liu G.Q., Xia H., Liu Y., Lu M.H. (2014). Conductivity reconstruction algorithms and numerical simulations for magneto–acousto–electrical tomography with piston transducer in scan mode. Chin. Phys. B.

[14] Dai M., Chen X., Chen M., Lin H.M., Li F.F., Chen S.P. (2018). A Novel Method to Detect Interface of Conductivity Changes in Magneto-Acousto-Electrical Tomography Using Chirp Signal Excitation Method. IEEE ACCESS.

[15] Dai M., Xiao X., Chen X., Lin H.M., Wu W.Q., Chen S.P. (2016). A low-power and miniaturized electrocardiograph data collection system with smart textile electrodes for monitoring of cardiac function. Aust. Phys. Eng. Sci. Med..

[16] Roth B.J., Schalte K. (2009). Ultrasonically-induced Lorentz force tomography. Med. Biol. Eng. Comput..

[17] Kunyansky L. (2011). A mathematical model and inversion procedure for magneto-acousto-electric tomography. Inverse Probl..

[18] Guo L., Liu G.Q., Xia H. (2015). Magneto-Acousto-Electrical Tomography with Magnetic Induction for Conductivity Reconstruction. Chin. Phys. Lett..

[19] Renzhiglova E., Ivantsiv V., Xu Y. (2010). Difference frequency magneto-acousto-electrical tomography (DF-MAET): Application of ultrasound-induced radiation force to imaging electrical current density. IEEE Trans. Ultrason. Ferroelectr. Freq. Control.

[20] Sun Z.S., Liu G.Q., Xia H., Catheline S. (2018). Lorentz Force Electrical Impedance Tomography Using Linearly Frequency-Modulated Ultrasound Pulse. IEEE Trans. Ultrason. Ferroelectr. Freq. Control.

